# Protecting the Herd: Why Pharmacists Matter in Mass Vaccination

**DOI:** 10.3390/pharmacy8040199

**Published:** 2020-10-26

**Authors:** Lois Lee, Gregory M. Peterson, Mark Naunton, Shane Jackson, Mary Bushell

**Affiliations:** 1 Discipline of Pharmacy, Faculty of Health, University of Canberra, Bruce, ACT 2617, Australia; u3223347@uni.canberra.edu.au (L.L.); g.peterson@utas.edu.au (G.M.P.); mark.naunton@canberra.edu.au (M.N.); 2 School of Pharmacy and Pharmacology, University of Tasmania, Hobart, TAS 7000, Australia; shane.jackson@utas.edu.au

**Keywords:** vaccine, COVID-19, herd immunity, Australian healthcare, pandemic, immunisation, pharmacist, pharmacy

## Abstract

Background: The COVID-19 pandemic is ongoing. The unprecedented challenges worldwide implore the urgent development of a safe and effective COVID-19 vaccine. Globally, pharmacists have been delivering important public health services as part of the COVID-19 response. It remains to be seen what role they will play once a vaccine is available. This review examines herd immunity and the potential role of the pharmacy profession in mass vaccination against COVID-19, particularly within the Australian context. Aim: A literature review was conducted to review the global development of COVID-19 vaccines, and the Australian healthcare workforce capability and existing policy for mass vaccination and the potential role of the pharmacist. Method: ScienceDirect, Scopus, The National Centre for Biotechnology Information (NCBI), Wiley Online Library, PubMed, and Google Scholar were used to search for relevant literature using keywords COVID-19, vaccines, immunisation, herd immunity, pandemic, pharmacist and Australian healthcare. Results: A large portion of the literature was journal articles, and information from governmental and international bodies such as the World Health Organisation were often referenced. Over 20 million Australians need to be immunised through vaccination or acquire immunity through natural infection for the country to achieve herd immunity for COVID-19. When examining state and territory pandemic plans, pharmacists are underutilised. Modifying legislation to allow pharmacists to administer approved COVID-19 vaccines will enable a trained and skilled workforce to be deployed to increase the rate of mass vaccination. Conclusion: In preparation for a successful COVID-19 vaccine, the Australian Government must consider various elements in their vaccination policy. This includes the estimated herd immunity threshold, methods of vaccine delivery, vaccine clinic locations, staffing arrangements and training, and strategies for vaccine prioritisation. Pharmacists can and should play a key role in the roll out of mass COVID-19 vaccination.

## 1. Introduction

COVID-19 is an infectious disease caused by a novel coronavirus, severe acute respiratory syndrome coronavirus 2 (SARS-CoV-2). The outbreak began in Wuhan, China, in December 2019 and a global pandemic was declared on 11 March 2020 by the World Health Organisation (WHO) [1]. As of 24 October 2020, WHO reported more than 42 million confirmed cases of COVID-19 worldwide, including more than 1.1 million deaths [2].

COVID-19 is highly contagious and may be asymptomatic or have impacts extending to severe respiratory distress, pneumonia and death, with the current mortality rate at approximately 6.9% [3]. The global focus to mitigate the disease is on the development of a vaccine, as vaccination is one of the most effective protection strategies against viral infections [3].

## 2. Vaccines and Herd Immunity: Background

### 2.1. Vaccination and Immunisation

Vaccination works by exposing individuals to modified antigens in order to develop immunological memory before encountering live pathogens [4]. It can provide long-lasting immunity and has proven to be the most effective approach to reduce the incidence of vaccine-preventable diseases [5]. Vaccination has successfully eradicated smallpox and it continues to provide protection against infectious diseases including pneumonia, measles, mumps, rubella, tetanus, polio, pertussis, varicella, hepatitis B and *Haemophilus influenzae* serotype b (Hib) [6].

### 2.2. “Herd Immunity”

“Herd immunity” is a term used to describe a population-scale immunity barrier, which can be achieved when a threshold of the population acquires effective immunity, by vaccination or natural infection, to the pathogenic bacteria or virus [7]. It protects the unvaccinated population by reducing the number of susceptible hosts to a level less than the threshold for transmission [8]. Understanding how vaccination programs work in the context of herd immunity provides insights into potential national and global COVID-19 vaccination strategies and campaigns [9].

The simple epidemic model (SIR model) provides a mathematical description of the spread of an infectious disease in a large population [10]. It consists of three categories of individuals: susceptible (S), infectious (I) and recovered/removed via vaccination (R). The R group needs to be sufficiently large to result in the infection not spreading, and therefore the achievement of herd immunity in the population. The number of people needed to be in the R group is determined by the infectivity of the disease, which is expressed through the basic reproduction number (R_0_) for infections [9]. R_0_ is the average number of secondary/new infections generated by a single infectious individual in a completely susceptible population [11]. R_0_ is dependent on the exponential growth rate of an outbreak and additional factors, such as the latent period (the interval between exposure to infection and the onset of the period of communicability) and the infectious period [12]. A higher value of R_0_ is associated with more infectious disease cases [9]. If R_0_ is less than one, a single infectious individual will infect less than one susceptible person and this will likely lead to the eradication of the disease.

The proportion of people who need to be in the R group, for herd immunity, can be calculated using this equation:

$$Threshold=1-\frac{1}{R_{0}}$$

For example, if the R_0_ for influenza is 1.3, the equation would be:

$$Threshold\;\left(influenza\right)=1-\frac{1}{1.3}=0.23\;or\;23\%$$

This would mean that at least 23% of the population needs to be in the R group for the R_0_ of influenza to become less than one, and therefore to prevent the spread of the infection [9,13].

### 2.3. Smallpox

Smallpox is caused by the variola virus and is transmittable from person to person by the inhalation of respiratory droplets or contact with infected material on mucous membranes [14]. The smallpox vaccine provided both cellular and humoral immunity to variola virus, with a high level of protection persisting 5 years after vaccination and partial immunity for 10 years or more [15]. The global smallpox eradication campaign adopted a herd immunity strategy to mass vaccinate the global population and achieve 80% vaccine coverage [16]. Following a successful eradication campaign using vaccinia virus vaccines during the 1970s, the WHO officially declared the eradication of smallpox in 1980 [17].

The R_0_ for smallpox was 4.9, indicating that a single individual infected with smallpox could infect 4.9 susceptible persons.

$$Threshold\;\left(smallpox\right)=1-\frac{1}{4.9}=0.8\;or\;80\%$$

The equation shows that 80% of the global population needed to be in the R group (to be recovered/removed via vaccination) for the R_0_ of smallpox to become less than one and thereby achieve herd immunity and eradication of the infection. The successful global campaign reflected this [17].

### 2.4. COVID-19

In the context of herd immunity, the estimated basic reproductive number, R_0_, for COVID-19 falls within the range of two to six [11]. Early research based on the initial 425 confirmed cases and their transmission in Wuhan, China estimated the R_0_ as approximately 2.2 [18]. However, considering recent research, the R_0_ figure is estimated to be higher, at 5.7, and therefore this figure is best used to inform herd immunity estimates [12]. If the threshold equation is applied with the estimated R_0_ number of 5.7, this would indicate that 82% of the population would have to be immune to achieve herd immunity.

$$Threshold\;\left(COVID-19\right)=1-\frac{1}{5.7}=0.82\;or\;82\%$$

International Federation of Pharmaceutical Manufacturers and Association (IFPMA) chief Thomas Cueni’s statement was in alignment with this figure, as he said that to create herd immunity against COVID-19, 80% of the population must be immunised [19].

However, researchers, policy makers and governments must consider that herd immunity only provides a rough guide to the approximate immunity threshold in a country’s population. There are more complexities in real-word populations, and other epidemiological and immunological factors, such as population structure, variation in transmissibility, factors of inter-individual heterogeneity, and waning immunity, must all be considered when discussing the guide to herd immunity within populations [11].

## 3. COVID-19 Vaccines: Global Landscape

Table 1 summarises the global landscape of COVID-19 candidate vaccines (WHO, 2020). There are many different vaccine formulations undergoing testing [20]. As of 19 October 2020, there are 44 candidate vaccines undergoing clinical trials and 142 candidate vaccines in preclinical evaluation [20]. Viral vectored vaccines, inactivated vaccines and RNA (mRNA) vaccines are in Phase 3 clinical trials (Table 2).

### 3.1. UK Landscape

The University of Oxford/AstraZeneca’s COVID-19 vaccine ChAdOx1 nCOV-19 (AZD1222) is currently in Phase 3 of its development. A randomised, double-blind, placebo-controlled multicentre trial with 30,000 participants is examining the efficacy, safety, tolerability and reactogenicity of two intramuscular (IM) doses of the vaccine in adults [24].

In the pre-clinical phase, the ChAdOx1 nCOV-19 vaccine was found to be immunogenic and protective against pneumonia in rhesus macaque (monkeys). In this species, the vaccine prevented damage to the lungs upon high-dose challenge with COVID-19 [25]. Phases 1 and 2 found that ChAdOx1 nCOV-19 was safe, tolerated and immunogenic, and elicited both humoral and cellular responses against COVID-19 in healthy individuals aged 18–55 years [24].

In the Phase 3 trial, vaccine efficacy in diverse populations and groups, such as older age groups, healthcare workers and those at highest risk, will be determined [24]. The estimated primary completion date is 2 December 2020 and the estimated study completion date is 5 October 2022 [26]. However, setbacks in the timeline are expected. A temporary pause was placed on the trial in early September 2020 after a participant suffered an adverse reaction. An independent review cleared recommencement of the trial after three days.

### 3.2. Australian Landscape

Australian researchers at the University of Queensland (UQ) are involved in the global effort to develop a COVID-19 vaccine. The UQ-CSL V451 vaccine commenced testing in a Phase 1 clinical trial in July 2020. The adjuvanted SARS-CoV-2 Sclamp protein subunit vaccine (UQ-1-SARS-CoV-2-Sclamp) vaccine is being evaluated for safety and immunogenicity using a randomised, double-blind, placebo-controlled, dosage-escalation, single-centre study. In Phase 1 the vaccine is being tested in healthy adults, aged between 18 and 55 years [27]. The estimated primary completion date for Phase 1 is October 2020 and the estimated study completion date is September 2021 [26].

Australia has also acquired an agreement with the University of Oxford/AstraZeneca. On 7 September 2020, Prime Minister Scott Morrison announced that the Australian Government has entered into a AUD 1.7 billion supply and production agreement with pharmaceutical companies such as the University of Oxford/AstraZeneca and the University of Queensland/CSL. Morrison announced that if the vaccines trials are successful, with the vaccine proven to be safe and effective and meeting all necessary regulatory requirements, the Australian population will gain free access to more than 84.8 million vaccine doses [28]. Early access to 3.8 million doses of the University of Oxford vaccine could occur in January and February 2021 [28]. However, setbacks could be caused by factors such as manufacturing challenges, insufficient sampling of population groups and unanticipated adverse reactions of the candidate vaccine [29].

## 4. Australian Healthcare Workforce Capability

Once a safe and effective vaccine is developed and planned for supply to the Australian population, policy makers must consider various elements in the successful rollout of the vaccine. Among many, one of the most significant elements to consider is the Australian healthcare workforce capability for mass vaccination.

The resident population of Australia is approximately 25.7 million [30]. If the threshold of 82% of the population needing to be immune to achieve herd immunity is applied, this would imply that about 21 million Australians would need to be immunised through vaccination or acquire immunity through natural infection to achieve herd immunity.

The NSW Health Influenza Pandemic Plan Policy Directive (PD2016_016) provides guidance for NSW Health staff and agencies to effectively prepare and respond to an influenza pandemic. Using Policy Directive 2016_016 as a guideline for the COVID-19 vaccine rollout, Local Health Districts (LHDs) and Specialty Health Networks (SHNs) will be responsible for developing strategies in providing the vaccination to the public within their district and a staff vaccination programme [31].

In terms of methods of vaccine delivery, Policy Directive 2016_016 states that LHDs/SHNs must collaborate with local health service and community providers to plan for appropriate models of delivery that meet the needs of the population, in accordance with NSW Ministry of Health guidelines. The vaccine clinic locations must allow for adequate crowd control, patient flow and space to facilitate patient assessment, vaccination and observation. Considering the location criteria, vaccination clinics may take form in general practice clinics, community centres (e.g., schools, sporting clubs) or through Aboriginal Community Controlled Health Services [31]. Community pharmacies with treatment rooms should also be considered as appropriate locations for COVID-19 vaccination.

In addition to a suitable location, an adequate number of trained immunisers is required to facilitate mass vaccination. Vaccines have traditionally been administered by a healthcare worker via needle and syringe injection during mass immunisation campaigns [32]. Vaccination is an accepted scope of practice for medical doctors and appropriately trained registered and enrolled nurses, midwives, aboriginal health workers and pharmacists.

The question of who can administer the COVID-19 vaccines currently differs between Australian states and territories [33]. While the Queensland government has passed legislation that will enable trained pharmacists to administer approved COVID-19 vaccines, to date, there has been no indication that other states and territories will amend regulations. When examining state and territory pandemic plans, pharmacists are underutilised. The peak professional pharmacy organisations are advocating that legislation across all states and territories should be amended to enable pharmacist-administered COVID-19 vaccination, well in time for mass rollout [34]. Doing so will enable a trained workforce to be deployed, increasing the rate of vaccination uptake so that the country can more rapidly achieve herd immunity.

Community pharmacies are recognised as the most accessed and most accessible health destination [35]. There are currently 5,762 community pharmacies located across Australia. Data show that 95% of people living in metropolitan areas and 65% of people living in rural areas live within 2.5 km of a community pharmacy [36]. Pharmacies play a critical role in connecting the Australian public with health resources, particularly in rural and remote areas.

During the COVID-19 pandemic, despite emerging challenges, internationally and nationally pharmacists are playing a key role in community and public health [37]. While many medical centres closed or went online, pharmacies remained open and pharmacists continued to deliver services to the public and enable continuity of medication supply. Many pharmacies also adopted digital technology and telehealth, and expanded services to include home delivery and outreach services [38,39]. In addition, pharmacists are involved in the mitigation of COVID-19, through screening, disease prevention education, supply of personal protective equipment, and point of care COVID-19 testing in some jurisdictions [37,40,41]. The willingness of pharmacists to step up and serve their community above and beyond expectation during COVID-19 pandemic, is evident.

Australian pharmacists have long played a key role in vaccine advocacy, education and distribution [40]. Since 2014, Australian pharmacists have been administering vaccinations to adults. From 2020, legislation across all jurisdictions had been modified to enable appropriately trained pharmacists to administer vaccinations to children, aged 10 years and over [41,42,43,44]. In recent years, legislation has been modified to enable pharmacists to administer vaccinations in mobile and outreach settings, and continuous education has been delivered to enable optimal service delivery [45,46]. Despite the trend for jurisdictional regulations to be amended to extend the types of vaccines a pharmacist can administer, the age of eligible patients, and locations in which they can be administered, there is a lack of uniformity across the nation.

While pharmacist-administered vaccinations should not be limited to the four walls of a pharmacy (e.g., they have been provided within aged care facilities [47]), it should be highlighted that most community pharmacies double as vaccination clinics; that is, they adhere to mandated vaccination service area facility and equipment requirements. Consistent with other authorised immunisers, pharmacist vaccinators are trained and required to upload information about each vaccine administered to the national register, Australian Immunisation Register (AIR). AIR collates data about the individual who received the vaccine, the health professional administering the vaccine, the date, time, type of vaccine and injection site used for administration. It also records that informed consent was obtained. Ensuring a consistent recording system for vaccine details is critical, as it is likely that the COVID-19 vaccines will be administered as part of a series, and dosing intervals and current vaccination status need to be easily identifiable to optimise vaccination coverage. Individuals can download information uploaded to the AIR via their individual immunisation history statements, and reminder notifications are also generated from the data collated in the AIR; again, the latter will be important given it is likely that several doses of the COVID-19 vaccines will be required.

A growing body of evidence shows that amending policy to enable pharmacist-administered vaccination has safely increased vaccination accessibility and uptake [48,49,50,51,52,53,54,55]. In 2020, during the COVID-19 pandemic, Australian pharmacists administered almost 1 million influenza vaccinations, a 300% increase on the year prior [34,56]. This equates to an average of 173 vaccines per pharmacy, or 14 influenza vaccinations each pharmacy per week using a 12-week influenza vaccination season. This undeniably reduced the risk of influenza morbidity and mortality. It also provides evidence that pharmacist workflow can accommodate an increased demand.

Indeed, pharmacists are preparing to be involved in the role out of the COVID-19 vaccines if called upon. Vaccination training programs to credential pharmacists, did not cease during the pandemic [57]. The peak professional organisations encouraged pharmacists who were not yet credentialed to complete vaccination training [58]. Vaccination training providers, such as the Pharmaceutical Society of Australia (PSA) and the Pharmacy Guild of Australia (PGA), and pharmacy schools continued to offer training to increase workforce capacity. Studies show that pharmacists are motivated to administer vaccines and that service delivery is linked with greater job satisfaction, and recognition as a health professional [59].

When rolling out a mass immunisation campaign, it is important to capture all Australians. For this reason, the hours of vaccination should also not be reduced to business hours. Most community pharmacies are open after-hours and on weekends, providing options to the public and increasing the accessibility of vaccination services [36].

## 5. Australian Government Support

Australia has published the Australia Health Sector Emergency Response Plan for Novel Coronavirus (COVID-19) to outline the national approach in response to COVID-19. The National Coronavirus Vaccination Policy and National Novel Coronavirus Immunisation Program will become available once the Australian Government has assessed and approved a customised vaccine [60].

Along with the National Coronavirus Vaccination Policy and National Novel Coronavirus Immunisation Program, adequate funding for the immunisation campaign from the Australian Government is essential. For the United States, the current estimate suggests that the Congress and executive branch will need to establish 7300 community vaccination clinics, appropriate USD 10 billion for community vaccination clinics and invest in a massive vaccination campaign [61]. No estimates are currently available for the Australian context.

There is a need for the membership of the Australian Technical Advisory Group on Immunisation (ATAGI) to include pharmacy sector expertise, noting that there is experience in general practice, nursing, public health and infectious diseases, amongst others. For Australian pharmacists to be actively considered in the implementation of COVID-19 vaccination plans, representation in ATAGI is a vital first step [62].

## 6. Vaccine Prioritisation

The rollout of COVID-19 vaccines will most likely be in stages and will require a vaccine prioritisation policy. Decisions and principles surrounding vaccine prioritisation for states and territory jurisdictions will be made by National Cabinet, based on recommendations from the Australian Health Protection Principle Committee. Decisions will be informed by the best available scientific evidence and the assessment of relative risk of vulnerable groups [60]. The Australian Commonwealth Department of Health will coordinate distribution to states and territories, and state and territory Ministries of Health will coordinate the distribution of the vaccine to nominated vaccine dispensers, including LHDs/SHNs and general medical practitioners.

Vaccination against COVID-19 utilising pharmacists should be no more or less costly than utilising nurse immunisers or GPs, in that they should receive the same reimbursement for service delivery as those other health professionals. What it does do is expand reach and access, when timeliness and capacity are going to be paramount. It also leverages off an existing network of immunisers with supply mechanisms via wholesalers. There may be cost savings to the government associated with a more rapid roll out of mass vaccination (e.g., reduction in hospitalisation costs, work and school absenteeism) which will enable faster economic recovery.

Since the current data on COVID-19 suggests that mortality is strongly associated with age, this assumption should form the basis for policies involving vaccine prioritisation [61]. The global consensus in vaccine prioritisation suggests that vulnerable people and essential workers, including those administering the vaccine, should be the first to receive the vaccine. Vulnerable people include those with poorer health, who are older and/or with comorbidities such as diabetes or high cardiovascular risk. Essential workers include police officers, pharmacists, medical doctors and nurses in immediate patient care [61].

## 7. Conclusions

The COVID-19 pandemic has posed unprecedented challenges and impacts worldwide. The current global focus is on developing a COVID-19 vaccine, and it is a fast-moving space with 44 candidate vaccines undergoing clinical trials as of 24 October 2020. Australia’s existing agreements with two COVID-19 vaccine developers aim to provide the Australian population with more than 84.8 million vaccine doses in the next couple of years. In preparing for mass vaccination in Australia, it is critical that the Australia Government considers Australia’s healthcare workforce capability. This includes aspects of immunisation threshold, vaccine delivery, vaccine clinic locations and hours of operation, staffing arrangements, vaccine administration training, and strategies for vaccine prioritisation.

To date, the COVID-19 pandemic has highlighted the importance of pharmacies and pharmacists to the Australian healthcare system and public health. It has revealed new roles that pharmacists can fulfil during a public health crisis. Trained pharmacist vaccinators, across all Australian jurisdictions, should be included in the health workforce planning to enable quick and large-scale COVID-19 vaccination in order to achieve herd immunity.

## Figures and Tables

**Table 1:** COVID-19 candidate vaccines, as of 27 September 2020.

Developer	Type of Candidate Vaccine	Vaccine Platform	Clinical Trial Phase
University of Oxford/AstraZeneca	ChAdOx1-S	Non-Replicating Viral Vector	Phase 3
CanSino Biological Inc./Beijing institute of Biotechnology	Adenovirus Type 5 Vector	Non-Replicating Viral Vector	Phase 3
Gamaleya Research Institute	Adeno-based (rAd26-S+rAd5-S)	Non-Replicating Viral Vector	Phase 3
Sinovac	Inactivated	Inactivated	Phase 3
Wuhan Institute of Biological Products/Sinopharm	Inactivated	Inactivated	Phase 3
Beijing Institute of Biological Products/Sinopharm	Inactivated	Inactivated	Phase 3
Moderna/NIAID	LNP-encapsulated mRNA	RNA	Phase 3
BioNTech/Fosun Pharma/Pfizer	3 LNP-mRNAs	RNA	Phase 3
Novavax	Full length recombinant SARS CoV-2 glycoprotein nanoparticle vaccine adjuvanted with Matrix M	Protein Subunit	Phase 2b
Anhui Zhifei Longcom Biopharmaceutical/Institute of Microbiology, Chinese Academy of Sciences	Adjuvanted recombinant protein (RBD-Dimer)	Protein Subunit	Phase 2
Curevac	mRNA	RNA	Phase 2
Institute of Medical Biology, Chinese Academy of Medical Sciences	Inactivated	Inactivated	Phase 1/2
Research Institute for Biological Safety Problems, Rep of Kazakhstan	Inactivated	Inactivated	Phase 1/2
Inovio Pharmaceuticals/International Vaccine Institute	DNA plasmid vaccine with electroporation	DNA	Phase 1/2
Osaka University/AnGes/Takara Bio	DNA plasmid vaccine + Adjuvant	DNA	Phase 1/2
Cadila Healthcare Limited	DNA plasmid vaccine	DNA	Phase 1/2
Genexine Consortium	DNA Vaccine (GX-19)	DNA	Phase 1/2
Bharat Biotech	Whole-Virion Inactivated	Inactivated	Phase 1/2
Janssen Pharmaceutical Companies	Ad26COVS1	Non-Replicating Viral Vector	Phase 1/2
Kentucky Bioprocessing, Inc.	RBD-based	Protein Subunit	Phase 1/2
Sanofi Pasteur/GSK	S protein (baculovirus production)	Protein Subunit	Phase 1/2
Arcturus/Duke-NUS	mRNA	RNA	Phase 1/2
ReiThera/LEUKOCARE/Univercells	Replication defective Simian Adenovirus (GRAd) encoding S	Non-Replicating Viral Vector	Phase 1
Clover Biopharmaceuticals Inc./GSK/Dynavax	Native like Trimeric subunit Spike Protein vaccine	Protein Subunit	Phase 1
Vaxine Pty Ltd./Medytox	Recombinant spike protein with Advax™ adjuvant	Protein Subunit	Phase 1
University of Queensland/CSL/Seqirus	Molecular clamp stabilised Spike protein with MF59 adjuvant	Protein Subunit	Phase 1
Medigen Vaccine Biologics Corporation/NIAID/Dynavax	S-2P protein + CpG 1018	Protein Subunit	Phase 1
Instituto Finlay de Vacunas, Cuba	RBD + Adjuvant	Protein Subunit	Phase 1
FBRI SRC VB VECTOR, Rospotrebnadzor, Koltsovo	Peptide	Protein Subunit	Phase 1
West China Hospital, Sichuan University	RBD (baculovirus production expressed in Sf9 cells)	Protein Subunit	Phase 1
Institute Pasteur/Themis/Univ. of Pittsburgh CVR/Merck Sharp & Dohme	Measles-vector based	Replicating Viral Vector	Phase 1
Imperial College London	LNP-nCoVsaRNA	RNA	Phase 1
People’s Liberation Army (PLA) Academy of Military Sciences/Walvax Biotech	mRNA	RNA	Phase 1
Medicago Inc.	Plant-derived VLP adjuvanted with GSK or Dynavax adjs.	VLP	Phase 1

**Table 2:** Key features of COVID-19 candidate vaccine types in Phase 3 clinical trials, as of 27 September 2020.

Type of Candidate Vaccine	Key Features
Viral Vectored Vaccines	Consist of recombinant virus (viral vector), in which the genome of the unrelated, harmless virus is used to deliver the antigen into human cells [21]
Inactivated Vaccines	Vaccine that contain inactivated virus that is inactivated by chemical, radiation or physical methods, which is no longer infectious [22]
RNA (mRNA)	Encodes a stable perfused form of Spike protein (antigen) and delivers to human cell. Once delivered, the human cell produces a vaccine antigen from the genetic code [23]
